# Amplification by PCR Artificially Reduces the Proportion of the Rare Biosphere in Microbial Communities

**DOI:** 10.1371/journal.pone.0029973

**Published:** 2012-01-11

**Authors:** Juan M. Gonzalez, Maria C. Portillo, Pedro Belda-Ferre, Alex Mira

**Affiliations:** 1 Institute of Natural Resources and Agrobiology, Spanish Council for Research, IRNAS-CSIC, Sevilla, Spain; 2 Genomics and Health Department, Center for Advanced Research in Public Health (CSISP), Valencia, Spain; Argonne National Laboratory, United States of America

## Abstract

The microbial world has been shown to hold an unimaginable diversity. The use of rRNA genes and PCR amplification to assess microbial community structure and diversity present biases that need to be analyzed in order to understand the risks involved in those estimates. Herein, we show that PCR amplification of specific sequence targets within a community depends on the fractions that those sequences represent to the total DNA template. Using quantitative, real-time, multiplex PCR and specific Taqman probes, the amplification of 16S rRNA genes from four bacterial species within a laboratory community were monitored. Results indicate that the relative amplification efficiency for each bacterial species is a nonlinear function of the fraction that each of those taxa represent within a community or multispecies DNA template. Consequently, the low-proportion taxa in a community are under-represented during PCR-based surveys and a large number of sequences might need to be processed to detect some of the bacterial taxa within the ‘rare biosphere’. The structure of microbial communities from PCR-based surveys is clearly biased against low abundant taxa which are required to decipher the complete extent of microbial diversity in nature.

## Introduction

Microorganisms play a major role in the functioning of biochemical cycles of elements [1] involving a large variety of microbial taxa. Although the use of culture-independent methods has greatly enhanced our understanding of microbial diversity [2], the microbial world remains largely unexplored [3], [4]. The current view of microbial diversity suggests that it is larger than previously expected and too large to be experimentally approached [5], [6]. Current estimates of microbial abundance and diversity in nature suggest, for example, the existence of 10^4^–10^6^ different microorganisms per gram of soil and a total number of around 10^10^ microbes g^−1^ [5], [7], [8]. As well, a long list of novel microbial phyla has been discovered in the last years [6], [9], mainly as a result of carrying out molecular surveys of microbial communities in a variety of environments and habitats.

Current assessments of microbial communities are mainly based upon the PCR amplification products of the small subunit rRNA genes. From this information, microbial richness is generally approached and the microbial components of the environmental communities are detected [10], [11], [12]. However, biases to the original community composition have been reported during amplification by PCR which leads to deviations of product-to-template ratios [10], [13], [14]. Deviations of the actual information on microbial communities from PCR-based community assessments affect equally to the analyses carried out by amplicon pyrosequencing [11], [15], [16] and other sequencing and screening procedures involving PCR, such as cloning and Sanger sequencing [17], denaturing gradient gel electrophoresis (PCR-DGGE) [18], and terminal restriction fragment length polymorphisms [19], among other methods. This is a point of major importance that needs to be resolved to reach accurate estimates of microbial richness, i.e., alpha diversity, and an actual view of the structure of microbial communities although it has been considered to be less of a concern for beta diversity comparisons [5], [11], [12].

Several causes of bias during PCR amplification have been cited. Among them, the universality of primers used in the amplification reaction has been questioned [12] and primer mismatch [14] has been considered a source of discrimination during PCR amplification. The use of primers targeting different 16S rRNA gene zones has also lead to differential results [12], [20], [21]. Different DNA polymerases can also discriminate the amplification of specific templates through differential amplification efficiencies and the use of optimized annealing temperatures should also be considered [22], [23], [24]. Differences in template sequences such as GC content can induce discrimination during amplification [10], [22]. Amplicon length has also been shown to reduce diversity estimates at increasing lengths [11], [12], [25] since longer targets are amplified with lower efficiencies [11], [22]. The dilution of the DNA template has also been reported as a factor affecting negatively the detection of low abundant taxa [24], [26]. The presence of high abundant taxa in low complexity communities can also lead to inhibition due to the possibility of template annealing during the late cycles of the PCR amplification [27]. Cycle number has been reported to potentially induce changes in the product-to-template ratios during the assessment of microbial communities although the major effect of a too elevated cycle number has been reported to be an increase in the potential for generating chimeras [28], [29] which can result in overestimates of the actual diversity in the studied communities. Genome size and the number of copies of the 16S rRNA gene per genome of the different microorganisms composing a community is also a problem to quantify the species and abundance of microbes in the environment [30]. Despite the identification of numerous potential causes for biases during PCR amplifications, the mechanisms affecting most of these biases are not well understood.

Present knowledge assumes that microbial communities are composed by a relatively low number of high abundant microbial taxa and a high number of poorly represented taxa. This last fraction has been named the ‘rare biosphere’ [15], [31], [32]. This portion of the community is greatly affected by biases and discriminations during PCR-based microbial surveys. Abundant taxa are easily identified through PCR but the detection of the rare microorganisms is highly dependent on budget and sampling effort which dictate current estimates of microbial richness and evenness. Understanding how PCR amplifications affect the detection of these low abundant taxa is important to estimate sampling or sequencing efforts required to document on taxa represented by relatively low fractions within microbial communities. Solving this problem is essential to obtain an accurate view of the structure of microbial communities and to warrant an adequate performance of PCR-based microbial surveys and shotgun-based metagenomic methods [4].

A successful PCR depends on the amplification efficiency which dictates the final PCR product yield. A PCR proceeds according to the following equation [33]:
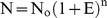
(1)where, N and N_o_ are the final and initial number of copies of the amplified target sequence, respectively; E is the efficiency of amplification ranging from 0 to 1; and n is the number of cycles of the amplification reaction. Maintaining the number of PCR cycles constant, the reaction depends on the amplification efficiency and the copies of the target DNAs.

Amplification efficiency, or fold amplification per cycle, is influenced by a number of factors including target sequence length, sequence base composition, primer sequences and specificity, buffer compositions, presence of PCR inhibitors in the template DNA solution, cycling conditions, and thermostable DNA polymerase. In order to determine the effect of specific factors on the potential biases caused during a PCR amplification, these variables must be reduced to simplify the problem to be studied. For instance, different sets of primers and amplicon lengths result in variations of the estimated microbial diversity [11], [12], [21]. During PCR amplification, the presence of a mixture of DNA sequences from the members of a community can result in a differential amplification as a consequence of competition between target sequences [34]. If a random amplification on every sequence present in the DNA mixture occurs, no significant differences in the product-to-template ratios should be observed. However, if a drift from randomness happens, a discrimination of some sequences could be expected. Understanding this phenomenon during the PCR might draw a direction for future normalization of amplification data aiming to obtain a realistic view of the structure of microbial communities.

The objective of this study is to determine whether the proportion of target sequences within a community could affect their detection through PCR-based methods from microbial communities. The study aims to assess whether low abundance taxa are being discriminated through PCR-based microbial community surveys and to analyze the dependence between amplification efficiency and the fraction that a specific sequence represents in a community. A quantitative, real-time, multiplex PCR amplification approach has been proposed to analyze the effect of the proportion of specific sequences during amplification. The potential influence of PCR amplification on the resulting view of microbial communities is analyzed through artificial and experimental communities.

## Methods

### Laboratory bacterial assemblages

Multispecies bacterial assemblages were prepared by combining the appropriated quantities of DNA from four bacterial species: *Bacillus subtilis* (strain 168), *Deinococcus radiodurans* (strain R1), *Escherichia coli* (strain K12), and *Pseudomonas aeruginosa* (strain PAO1). The bacterial genome size and the number of copies of the 16S rRNA genes present in each of their genomes are known (Table 1). The proportion of sequences in these DNA mixtures was considered as number of copies of the 16S rRNA genes from each bacterial species. Bacterial DNA was extracted using a conventional protocol based on bacterial lysis by a treatment with lysozyme followed by protease, phenol/chloroform extraction and ethanol precipitation. DNA concentration was determined using a nanodrop spectrophotometer (Nanodrop Technologies Inc., Wilmington, DE, USA) with triplicate measurements.

**Table 1:** Bacterial species used in this study including the number of 16S rRNA genes per genome and their genome length.

Bacterial species	Strain	16S rRNA genes per genome	Genome length (Mbp)
*Bacillus subtilis*	168	10	4.2
*Deinococcus radiodurans*	R1	2	3.2
*Escherichia coli*	K12	7	4.6
*Pseudomonas aeruginosa*	PAO1	4	6.3

### Experimental approach

The goal of these experiments was to assess the amplification efficiency for distinct bacteria present at different proportions in multispecies DNA templates. To determine the potential consequences for the detection of taxa during PCR-based microbial surveys, the designed experiments focused on reducing the number of variables. To reduce the potential effect of amplicon size on amplification, the same primer pair was used for the amplification of the bacterial species. The amplification of these four bacterial species was monitored simultaneously by quantitative, real-time multiplex PCR using species-specific Taqman probes (Table 2) in the same amplification tube. The total DNA, sum of the four bacterial species was kept constant in all the reactions (100 ng except if noticed otherwise). The amount of DNA from each bacterial species varied to cover a wide range from 0.01% to 100% of copies in the total DNA mixture.

**Table 2:** PCR primers and labeled probes utilized in the present study.

Primer/Probe	No. bases	Label^1^	Sequence (5′->3′)	Target	Reference
341F	17	None	CCT ACG GGA GGC AGC AG	Bacteria	Muyzer et al., 1993
518R	17	None	ATT ACC GCG GCT GCT GG	Bacteria	Muyzer et al., 1993
Bs473p	21	3′ BHQ1	GGT ACC GCC CTA TTC GAA CGG	*Bacillus subtilis*	This study
		5′ FAM			
Dr461p	22	3′ BHQ2	TCT GCC CTA AGG CTC TTT CGT C	*Deinococcus radiodurans*	This study
		5′ Cy5			
Ec469p	25	3′ BHQ1	TGA GCA AAG GTA TTA ACT TTA CTC C	*Escherichia coli*	This study
		5′ HEX			
Ps470p	24	3′ BHQ2	AAC AGC AAG GTA TTA ACT TAC TGC	*Pseudomonas aeruginosa*	This study
		5′ ROX			

1 BHQ, Blackhole Quencher.

### Detection of bacterial species

Quantitative, real time, multiplex PCR amplification was carried out with the QuantiTect Multiplex PCR kit (Qiagen GmbH, Hilden, Germany) on an iCycler iQ Real-Time PCR Detection System (Bio-Rad, Hercules, California). In order to avoid the potential effect of using different primers for amplifications from distinct bacteria, the same primer pair was used for the amplification of their 16S rRNA genes. The primers for the amplification of the 16S rRNA gene fragments were 341F and 518R (Table 2). Each Taqman probe targeted the amplified region of one of the bacterial species (Table 2). Each probe had a different fluorescent dye and quencher to simultaneously detect the amplification of the 16S rRNA gene fragments from each bacterial species (Table 2). Thermal conditions followed the manufacturer recommendations and consisted of a denaturation at 95°C for 15 min followed by 50 cycles with two temperature steps each: denaturation at 94°C for 60 s, and a step at 60°C for an annealing/extension period of 50 s plus an extension/data collection step for 40 s at the same temperature. Triplicate reactions were processed. Controls with a single bacterial species showed no cross detection in the different channels. Real-time amplification data were background subtracted by the iCycler iQ software. Quantification was performed following the sigmoidal non-linear curve-fitting procedure described by Rutledge [35] which allows the estimation of the amplification efficiency for each reaction. Non-linear curve fitting was performed with Sigmaplot 8.02 (Systat Software, Inc., London, UK). The relative amplification efficiency (E_r_) for each bacterial species and fraction of 16S rRNA gene copies was estimated as the fraction of the maximum amplification efficiency (E_T_) which was determined in reactions with DNA from only that specific species. Relative amplification efficiency values ranged between 0 and 1.

One-tailed F-test for differences between variances was used to check if significant improvement of curve fitting existed between two models as described by Gonzalez [36] and Sokal and Rohlf [37]. Original communities and those resulting after computational amplification following the proposed model were compared by the G-test of goodness-of-fit [37] to determine if significant differences existed between these communities.

### Analyses of artificial communities

Two examples of artificial communities were analyzed to approach the effect of the proportion of bacterial taxa on their detection through PCR-based surveys. The composition of these communities is indicated in Table 3. The original frequency of the OTUs (Operational Taxonomic Units) or taxa in these communities was compared to a final community obtained after computation of the expected amplification. The PCR was simulated assuming a random amplification according to equation 1, and following the model proposed in this study (see below) which relates relative amplification efficiency to the composition of the microbial community. A total of 20 cycles of amplification at an E_T_ of 0.9 were considered. Comparisons were presented as product-to-template ratios estimated as the quotient of the frequency of the detected OTUs in the final expected community (after amplification) divided by the frequency of the corresponding OTUs in the original community. Ratios below one indicate a discrimination against those OTUs or taxa. Rarefaction curves were constructed according to Hughes and Hellmann [38] for the original community and the expected communities after amplification. The Shannon-Weaver diversity index and evenness were estimated according to Shannon and Weaver [39] and Sheldon [40], respectively. The Simpson index was calculated as Simpson [41]. The number of OTUs detected in these communities after amplification was estimated by computing the process of 10^8^ sequences from the total pool of amplified sequences. The OTUs that were not detected after processing 10^8^ sequences were considered as taxa remaining undetected and consequently were not included in the estimates of diversity indices. The sequencing effort required to detect an OTU was approximated as the inverse of the likelihood of detecting at least one sequence corresponding to that OTU.

**Table 3:** Composition of artificial communities I and II and estimates of the sequencing effort expected to detect these taxa through PCR-based surveys according to the proposed model.

Community I			Community II		
No. of Taxa	Frequency	Sequencing effort^1^	No. of Taxa	Frequency	Sequencing effort^1^
2	0.294	<10	5	0.05	<10
1	0.196	2×10^4^	5	0.04	2×10^5^
1	0.098	8×10^5^	5	0.03	4×10^5^
1	0.049	3×10^6^	7	0.02	1×10^6^
1	0.0196	1×10^7^	15	0.01	3×10^6^
5	0.0098	2×10^7^	12	0.005	1×10^7^
			34	0.001	1×10^8^
			24	0.0005	ND^2^
			23	0.0001	ND^2^
			25	0.00005	ND^2^
			25	0.00001	ND^2^
			33	0.000005	ND^2^
			35	0.000001	ND^2^
**Total No. of taxa** = 11			**Total No. of taxa** = 248		

Maximum sequencing effort considered was the processing of 10^8^ sequences.

1 Approximated sequencing effort required to detect an OTU present at the indicated frequency through PCR-based surveys.

2 Taxa requiring a sequencing effort higher than 10^8^ sequences were considered to remain undetected (ND).

### Analyses of experimental communities

Samples from the oral cavity of two different persons were analyzed through a metagenomic study by direct pyrosequencing (without PCR amplification) and by an amplicon bacterial survey after PCR amplification. The 16S rRNA gene sequences obtained from these two approaches were used for the comparison of the experimental communities. The procedure followed for the metagenomic analysis has been previously described in detail [42]. The protocol for the bacterial survey based on 16S rRNA gene amplicons included amplification with universal primers 27F and 533R at an annealing temperature of 52°C and after 20 cycles of amplification, and subsequent pyrosequencing following McKenna *et al.* [43]. The sequence between 27F and 533R primers covered the hypervariable regions V1 and V2. Pyrosequencing for these two procedures was performed in a GS FLX machine with titanium chemistry (Roche, Basel, Switzerland). Taxonomic assignment at the genus level was performed against the ribosomal Database Project using an 80% bootstrap cut-off following Wang *et al.* [44]. Sequences shorter than 250 nucleotides and those with average sequence quality values below 20 were filtered out. It was observed that sequence quality diminished at the end of long sequences, thus all reads were trimmed at 400 pb, significantly increasing the average quality of the reads. Sequences with differences in the primer region were excluded from the analysis as well as sequences with more than 4 ambiguities in homopolymeric regions. Sequences with unidentified barcodes were removed from the analysis and barcodes differed in at least two nucleotides in order to reduce the possibility of missassigment [45]. Product-to-template ratios were estimated assuming metagenomic data were exempt from PCR biases and thus representing the closest approximation to the actual template from the microbial community. PCR amplicon sequencing data were assumed to potentially suffer from PCR biases and represented the estimate of diversity after PCR amplification. Rarefaction curves and diversity indices were obtained as above.

## Results

### Amplification as a function of the proportion of the sequence in a community

**Figure 1:**
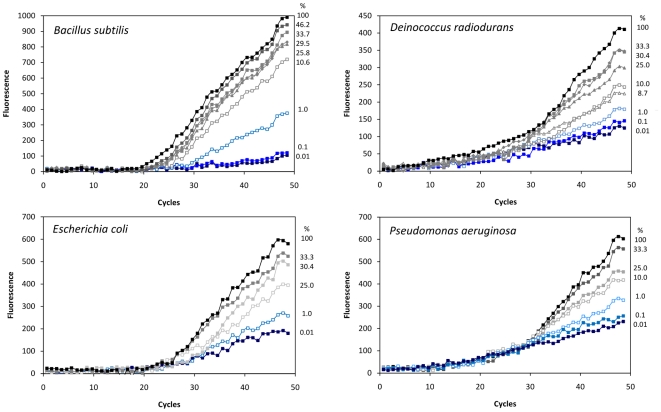
Real-time PCR amplification of DNA for four bacterial species. The plots correspond to amplification from different multispecies DNA mixtures representing different fraction of total DNA template, from 0.01% to 100%. The bacterial species are: *Bacillus subtilis*, *Deinococcus radiodurans*, *Escherichia coli*, and *Pseudomonas aeruginosa*. The percentage of the examined bacterial species for each reaction set is indicated on the right.

In order to detect individual bacterial species within multispecies DNA mixtures, species-specific Taqman probes were designed. Multiplex PCR amplifications were performed using the same primer pair for all targeted species. Variations of the proportion of the four species forming these DNA templates resulted in a series of amplification results for each bacterial species representing a wide range of fractions within the total template DNA. The amplification of individual bacterial species varied as a function of the fraction of the total copy number they represented in the community (Figure 1). When a bacterial taxon represented less than 1% of the target sequences, the amplification reaction barely generated any products corresponding to that species. Similar patterns are observed for each of the four species tested in the experiments.

Due to the different amplification efficiency observed for the distinct species, the results were normalized by calculating the relative amplification efficiency (E_r_) for each species and fraction of total multispecies DNA. Maximum amplification efficiencies (E_T_) estimated for the DNA from *B. subtilis*, *D. radiodurans*, *E. coli* and *P. aeruginosa* were 0.92, 0.40, 0.48 and 0.32, respectively. Analysis of the 16S rRNA gene sequences from these four bacterial species indicated that they perfectly matched the primer sequences and the amplification resulted in PCR products of 195 base pairs except for *D. radiodurans* that generated a 181 base pair product (including primer sequences).

### Relationship between relative amplification efficiency and the proportion of a species in a community

**Figure 2:**
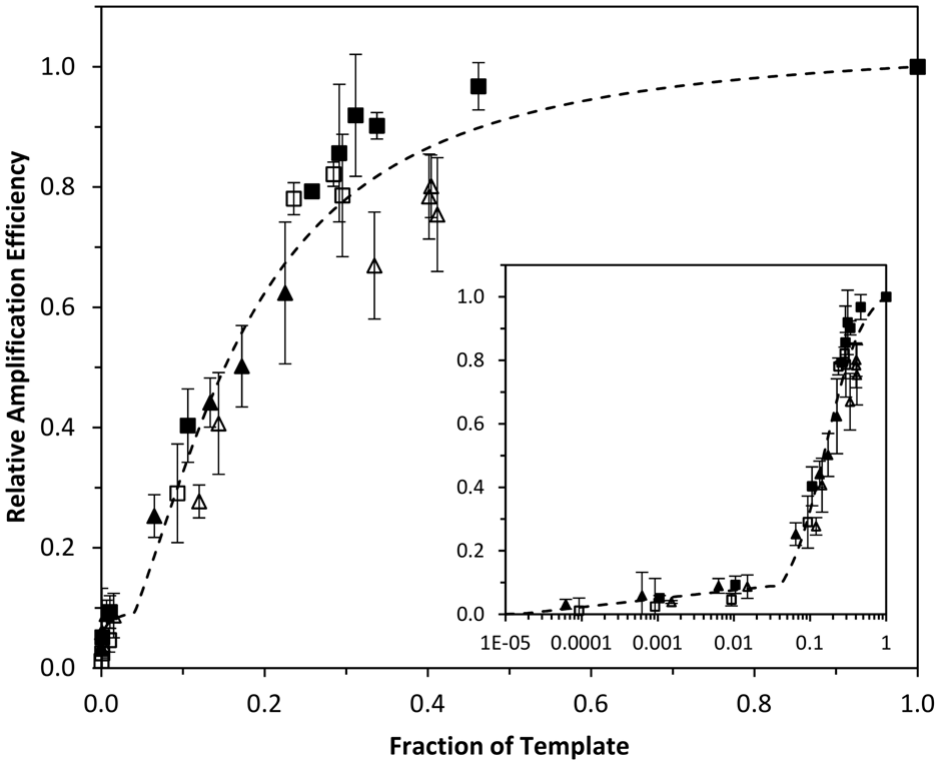
Relationship between the relative amplification efficiency of 16S rRNA genes from four bacterial species. *Bacillus subtilis* (black squares), *Deinococcus radiodurans* (white triangles), *Escherichia coli* (white squares), and *Pseudomonas aeruginosa* (black triangles), at different proportions within the community. Linear (A) and logarithmic (B) scales are shown to see the curve fitting for large and low initial proportions of the target sequences within the community.

The quantitative, real-time, PCR amplification curves allowed estimates of the relative amplification efficiencies for each bacterial species representing different fractions of the total DNA. The estimated relative amplification efficiencies for each bacterial species showed a dependence of the fraction they represented within the multispecies DNA templates. Similar pattern was observed for the bacterial species forming the DNA mixtures and were analyzed globally. Figure 2 shows a total of 34 data points and a plot of the sigmoidal relationship observed between E_r_ values and the fraction of total number of copies in the multispecies DNA.

The data points showed the best curve fitting to a Hill, three parameters, equation model (Equation 2) [46] expressing relative amplification efficiency (E_r_) as a function of the fraction represented by the copies of the 16S rRNA gene in the experimental, multispecies DNA mixtures. The equation model was:
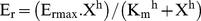
(2)where, X is the fraction of 16S rRNA gene copies corresponding to the studied species within a multispecies DNA template; E_r max_ is the maximum value that the relative amplification efficiency could reach which should be 1; K_m_ is the value of X corresponding to ½ E_r max_; and h is a modifier exponent shaping the curve and indicates the sharpness of the transition from the low to the high E_r_ values.

At the lowest fraction tested in this study, below E_r_ values of 0.109 (corresponding to a fraction of around 4.5% of total target sequences), the curve (Figure 2B) showed a lower slope than expected with the relationship observed for higher fraction values. The experimental data for the lowest range of fractions were approached by the equation:
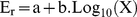
(3)where, a and b are the coefficients of the equation.

The best fitting results were observed using a composition of two expressions (equations 2 and 3), equation 2 for equal to and higher than 4.5% fractions (Figure 2) and a semi-logarithmic relationship for the lowest fractions (<4.5%; Figure 2B). These two equations collaborated to obtain a significantly better fitting (P<0.001) to the experimental results as tested by a one-tailed F-test [36], [37]. The estimated parameters (± standard deviations) showing the best fit to the data were as followed:
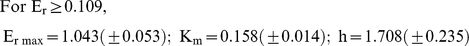


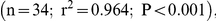


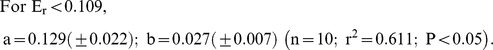
The bacteria representing fractions of the total DNA template lower than Km were poorly amplified during PCR reactions. Bacteria present in high proportions were easily detected through PCR-based surveys because they were amplified with high relative efficiencies. Bacteria representing fractions lower than 1% of the total community experienced minimum amplification during PCR.

### Comparisons of pre- and post-PCR using artificial communities

The proportion represented by a microbial taxon is essential for its detection during PCR-based surveys of microbial communities. From the results above, a model was built to estimate potential consequences of these results on the detection of microbial taxa from artificial communities. Two different communities are presented as examples (Table 3). Community I is formed by some highly abundant taxa (up to 30% of the total community) and relatively low diversity (a total of 11 taxa). Community II is a much highly diverse example composed by 248 OTUs that were distributed in frequencies from about 0.0001% to 5% of the total community. These communities were analyzed by a random amplification model and by the proposed model above (Figure 2). The original and random model showed identical results to the initial communities previous to amplification and there were no differences between random model results and the initial communities in the rarefaction curves, product-to-template ratio, and sampling efforts.

**Figure 3:**
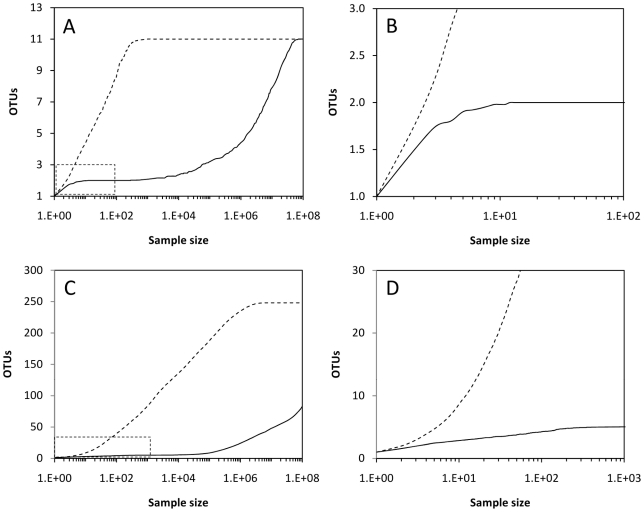
Rarefaction curves obtained for two different artificial communities. A, Community I with a total of 11 OTUs and C, Community II with a total of 248 OTUs. The curves for the initial community (black dashed lines) and post-PCR communities (solid lines) according to the proposed model (Figure 2) are shown. The portion of the curves included in the dotted squares are amplified on the right. B and D correspond to the lowest sampling sizes for A and C, respectively.

**Figure 4:**
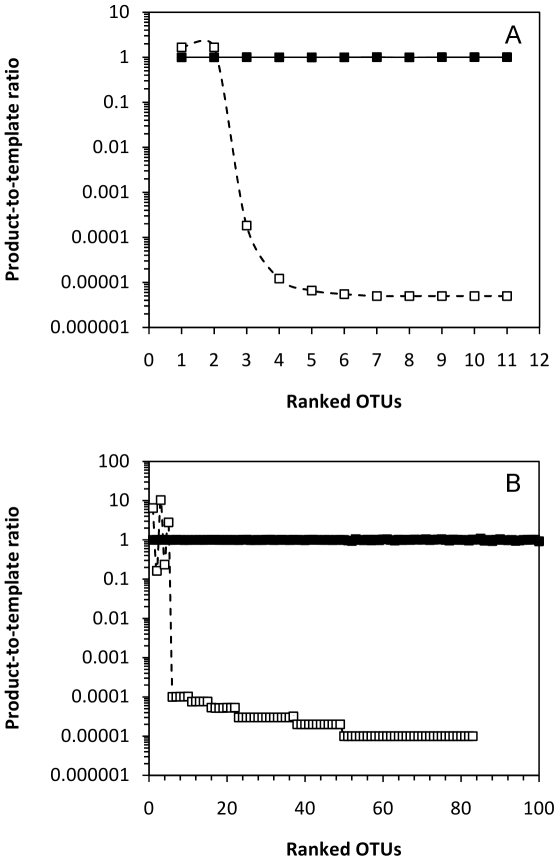
Product-to-template ratios *versus* OTU ranking for two artificial communities. Two artificial communities (I and II) are shown. They were estimated by following a random amplification (black squares) and the proposed model (Figure 2) (white squares) of the relative amplification efficiency as a function of the fraction that each OTU represents within the total community. A, Community I; B, Community II.

The model proposed in this study resulted in clear differences between the original and final community structure. Rarefaction curves (Figure 3) for community I showed marked differences. The rarefaction curve generated for the original community (and the random amplified community) showed a much steeper slope than the one obtained for the proposed model (Figure 3A) and only when the sampling effort increases considerably (>10^5^ processed sequences) the low abundant OTUs (<10%) start to be detected. With Community I, the product-to-template ratios decrease sharply for OTUs other than the most abundant (Figure 4A). In this case, the taxa represented at a fraction of ≤20% were clearly discriminated during amplification by, at least, a factor of 10^4^–10^5^. In this community where there are OTUs representing 30% of the community, the processing of over 10^4^ sequences is required to detect OTUs represented by 10–20% of the total community and increasing efforts are needed (over 10^7^ sequences) to detect those members accounting for 1–5% of the total community. Table 4 shows the estimates of various indices for the original Community I and the detected community after amplification followed the model proposed in Figure 2 and the randomly amplification model. The proposed model (Figure 2) induces low diversity index values as a result of the primary detection of the abundant taxa and a negative discrimination against the low proportion ones.

**Table 4:** Comparison of diversity indices estimated from two artificial and two experimental communities.

	Shannon	Evenness	Simpson	No. of taxa^1^
**Artificial Community I**				
Original community	1.719	0.093	0.776	11
Random amplification	1.719	0.093	0.776	11
Proposed amplification model	0.694	0.038	0.500	11
**Artificial Commmunity II**				
Original community	3.840	0.209	0.970	248
Random amplification	3.840	0.209	0.970	248
Proposed amplification model	1.075	0.058	0.609	83
**Experimental Community 1**				
Metagenome analysis	2.598	0.377	0.883	28
Amplicon analysis	2.522	0.366	0.904	17
**Experimental Community 2**				
Metagenome analysis	2.419	0.351	0.881	33
Amplicon analysis	2.466	0.358	0.876	28

For the artificial communities, the analysis includes the original communities, and the communities obtained after a theoretically random amplification and after simulating the proposed amplification model. For the experimental communities, results of the 16S rRNA analysis from metagenome analysis through direct sequencing (lacking amplification by PCR) and 16S rRNA amplicon analysis after PCR amplification are presented.

1 Number of taxa detected during the analyses. For the artificial communities 10^8^ sequences were screened. For the experimental communities around 10^3^ sequences were processed.

Community II also showed obvious differences between the rarefaction curves obtained for the original community (and the randomly amplified model) and the model proposed in this study (Figure 2). The process of over 10^5^ sequences was required to detect 10% of the community OTUs (Figure 3B). Even a maximum sampling of 10^8^ sequences was unable to detect all the members of the community, and only 83 out of 248 OTUs (33% of the community) were detected at this maximum sampling size (Figure 3B). In Community II, the OTUs representing lower fractions than 1% suffered a sharp discrimination as noticed by the product-to-template ratios below 10^−4^ for these taxa. The processing of 10^8^ sequences was only valid to detect a small portion of the members constituting Community II indicating that around 67% of the community would remain undetected after this survey. OTUs present in the original community at percentages of 0.05% or less could not be detected even at the highest sampling size (Table 3). The diversity indices estimated for the original Community II (Table 4) and the taxa detected from this community after amplification shows a reduction of the calculated indices as well as the detection of only a small fraction of the taxa constituting that community.

Interestingly, for both communities I and II, the rarefaction curves at relatively low sampling sizes (Figure 3B and 3D) show a false plateau at low sampling efforts. At increasing sampling sizes, the number of detected OTUs continues to increase. This double plateau could provide wrong impressions about the level of representation in the studied community.

### Comparison of experimental data from metagenome and amplicon surveys

**Figure 5:**
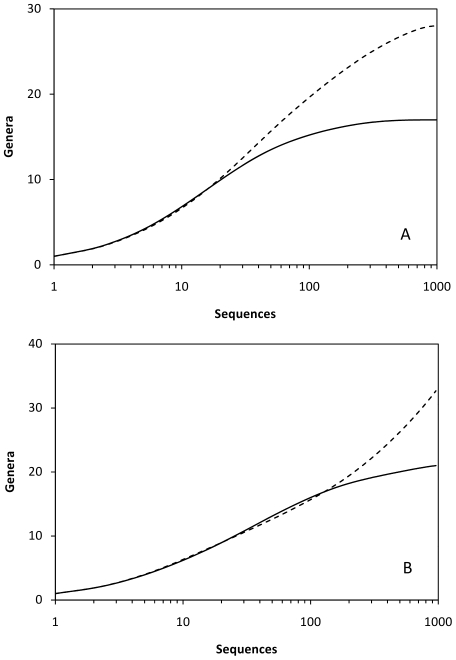
Rarefaction curves at the genus level obtained for two experimental microbial communities from the human oral cavity. The curves shown correspond to the results of 16S rRNA gene analyses from a metagenome study through direct sequencing (no PCR amplification) (black dashed lines) and from a microbial survey based on the 16S rRNA amplicon analysis after PCR amplification (solid lines). A, Experimental community 1; B, Experimental community 2.

**Figure 6:**
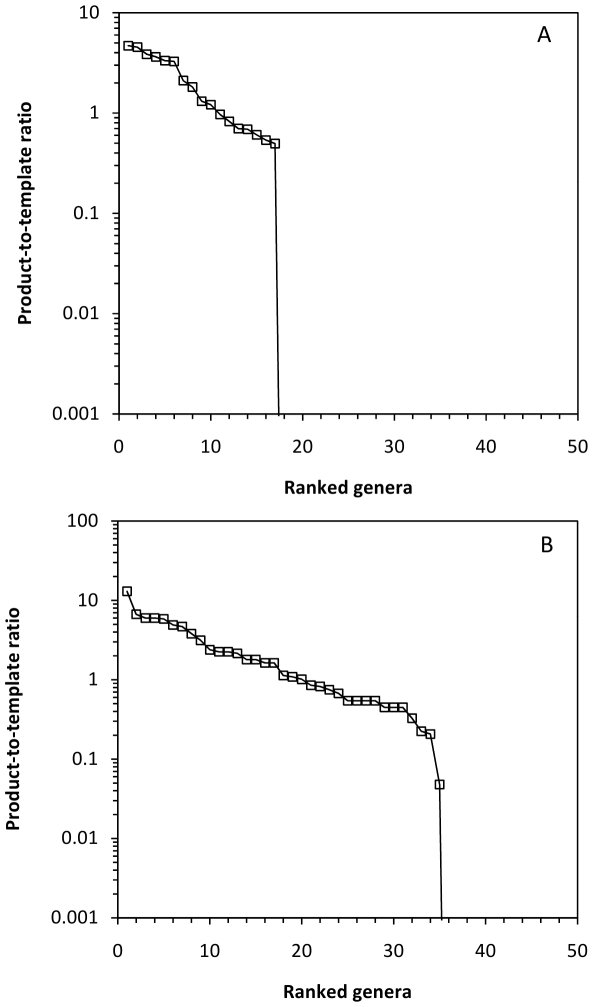
Product-to-template ratios *versus* ranked genera for two experimental communities. Two communities (1 and 2) from the human oral cavity were analyzed. The product was represented by the amplicon analysis data and the template was considered the results obtained from a metagenome analysis lacking PCR amplification. A, Experimental community 1; B, Experimental community 2.

When comparing experimental communities, the total diversity, or number of different OTUs, in the communities is unknown. A comparison of the rarefaction curves obtained from the metagenome data and from amplicon surveys resulted in differences in the expected number of OTUs present in the community through these two methodologies (Figure 5). Metagenome studies involved no PCR amplification and were assumed to represent the closest available data to the community of origin. The product (amplicon survey data) to template (metagenome data) ratios (Figure 6) showed the amplification of a limited number of relatively abundant OTUs. As suggested from Figure 5, the expected number of OTUs in the community was higher than that obtained from amplicon surveys. Some of the lower abundance OTUs remained undetected through PCR amplification analysis. Experimental communities 1 and 2 analyzed through PCR amplification resulted in a 39.3% (17 out of 28 OTUs) and 15.2% (28 out of 33 OTUs) of undetected taxa, respectively, with respect to metagenome analyses (Table 4). Thus, a sharp decrease in the product-to-template ratio represents a critical threshold for the detection of taxa through PCR-based techniques and has been detected in both artificial (Figure 4) and experimental (Figure 6) communities. Experimental communities were sequenced obtaining a total of 33 OTUs and 28 OTUs from the metagenome (shotgun sequencing) and 28 OTUs and 17 OTUs from 16S rRNA gene amplicons. The OTUs from amplicons were obtained from a total number of 230 and 2056 sequences from experimental communities 1 and 2, respectively. The total number of 16S rRNA gene fragments extracted from shotgun sequencing data were 664 and 1302 reads from experimental communities 1 and 2, respectively.

The data from metagenome and amplicon analyses from these communities can be accessed from MG-RAST (http://metagenomics.anl.gov/) under numbers 4447192.3 and 4474804.3, respectively, for community 1 and 4450726.3 and 4474910.3, respectively, for community 2.

## Discussion

Current understanding of the structure of microbial communities depends on the accurate assessment of diversity and the relative abundance of the components in the studied communities. However, microbial diversity remains difficult to estimate as a result of the large variety of different microorganisms present in environmental samples. Progress in DNA sequencing technology has permitted an increase in the scale of microbial community surveys [15], [16] although PCR amplification biases have been reported to skew the results and to hinder the detection of numerous microbial taxa [12], [21].

Despite the numerous factors reported to potentially cause amplification biases [11], PCR-based microbial surveys are the common approach in diversity studies [16]. Herein, quantitative, real-time, multiplex PCR using species-specific probes have been used to monitor the amplification of species-specific sequences in multispecies DNA mixtures. Our data show that the detection of 16S rRNA genes by PCR amplification depends on the proportion at which specific taxon sequences were present in the total multispecies DNA template. Moreover, a relationship could be established between the fraction of sequences from a specific taxon and its corresponding relative amplification efficiency. This pattern is independent of the bacterial species. As a consequence, sequences present at high abundance in a multispecies DNA template mixture are amplified with high relative amplification efficiencies. The low-frequency species show poor relative amplification efficiencies resulting in important under-representation during PCR-based microbial community surveys. These results explain a process of selection or discrimination of microbial taxa during PCR-based microbial surveys as a function of the representation of these taxa within the studied communities.

The use of combined procedures for DNA extraction has been proposed as a mean to determine more realistic microbial diversity estimates [21]. In our study, a standard DNA extraction procedure was performed with no further purification steps mimicking DNA extractions from environmental communities. Thus, the genomic DNA from different bacterial species was extracted although distinct quality DNA was obtained for each species. This influences the amplification of these bacterial species 16S rRNA genes as reflected by the differential amplification efficiency observed for these species. The values of absolute amplification efficiencies for the bacterial species DNA during this study are representative of experimental conditions from environmental samples. These values are in good agreement with the range reported by Arezi *et al.* [22] for different samples and DNA polymerases (from 36 to 88%). For instance, the frequent coextraction of humic acids with DNA inhibits PCR amplifications [47] causing a decrease of amplification efficiency [48]. Given that the detection of microorganisms through PCR depends on the amplification efficiency for the specific target sequences (according to equation 1), those species whose DNA is efficiently amplified will be easily detected and those amplified at low efficiencies will have low chance to be retrieved. The bacterial species used during this study showed perfect match to the sequences targeted by the PCR primers so the potential bias induced by sequence mismatches [12] was excluded in our case with laboratory bacterial assemblages. The experiments performed with oral bacterial communities strongly suggested that primer specificity was not of importance. *In silico* analysis of the 16S rRNA gene sequence fragments targeted by the primers used for amplification confirmed that most of the bacteria remaining undetected perfectly matched these primers. Only three of the genera undetected through PCR could potentially show some mismatches corresponding to under-represented species within these genera. These three genera were *Globicatella*, *Lonepinella* and *Phocoenobacter*. Different DNA extraction procedures could allow the lysis and purification of genomic DNA at the highest quality from each species. However, this is unfeasible for complex microbial communities. Improving the quality of the extracted DNA for those species presenting difficulty during the extraction (e.g., those producing excessive exopolymeric substances) would be desirable. The combination of different DNA extraction procedures is a recommendable approach to achieve a more realistic view of the microbial diversity (alpha diversity) in a specific environment.

Analysis performed with artificial communities showed that PCR amplification of sequences in multispecies communities or DNA templates favors the detection of the highly represented and acts against the species present at low frequencies. These results confirm significant skews in the detection of microbial taxa during PCR-based community surveys. Consequently, the detection and study of the microorganisms belonging to the ‘rare biosphere’ [15], [31] is unfavoured by PCR-based methodologies and so our current understanding of microbial diversity and the community structure of environmental systems is uncertain and clearly biased.

To understand the structure of microbial communities there are two major parameters that need to be determined: species richness and the fraction of the total community represented by each taxa. As suggested in previous studies [11], [12], [21] and confirmed in the present work, PCR-based microbial surveys affect both aspects. PCR-based community surveys can cause over 10^4^-fold under-representation of low abundant taxa as seen by a sharp reduction of product-to-template ratios for sequences present at frequencies of 1–5% and below depending on the composition of the microbial communities. These observations also support those by Engelbrektson *et al.* [12] indicating that rare populations were not reproducibly sampled despite studying relative simple communities. These results are also confirmed with experimental microbial communities showing that a number of taxa remained undetected through a PCR approach. The frequency of these taxa in the original microbial community is modified by the bias introduced during PCR amplification which affects the proportion of different microbial taxa and so, the obtained view of the studied community is to be distorted. By applying different scales of sampling size during sequence analysis our view of the potential level of diversity can be different. Relatively modest sequencing scales can suggest that a significant representation of the community has been achieved. Much higher sequencing scales could suggest otherwise. This artifact from PCR amplification during microbial community surveys can also present a confusing view of community composition and a wrong understanding of the ‘rare biosphere’. A relatively complete set of microbial taxa within the ‘rare biosphere’ can only be retrieved and detected by processing extremely high number of sequences although using PCR-based approaches an unrealistic view of the actual community will be obtained.

At present, PCR-based microbial surveys are the common procedure to investigate diversity and community structure of microorganisms in the environment and human-associated niches. The procedure involved in metagenomic approaches (i.e., shotgun-sequencing) does not require PCR amplification [16], [49]. However, the scale of such studies currently does not allow a complete detection of all taxa present in environmental samples. Consequently, PCR amplification approaches, despite their biases, are likely to remain in a foreseeable future as important tools to recover and assess microbial communities in the environment [21] and the scientific community must understand its limitations to correctly interpret microbial structure and diversity.

The results from this study have important implications for molecular studies of microbial communities. In agreement to previous reports on different causes of biases resulting from PCR-based microbial surveys [10]–[12], [21], [22], [27], [30], the present study suggests an explanation for some of the skews generated during PCR amplifications. These biases can be a result of the relationship existing between the relative amplification efficiency for specific sequences and the fraction of these sequences within multispecies DNA assemblages or microbial communities.

The results obtained from this study can be applied to better understand microbial community structure and the detection of 16S rRNA genes from environmental microbial communities and from the human microbiome. Gene surveys targeting other genes, besides the 16S rRNA gene, both of phylogenetic (e.g., *gyr*A, *rec*A, *rpo*A) and functional (e.g., *amo*A, *dsv*A, *mcr*A, *nif*H) interests will also be affected by PCR biases. So, surveys of microbial communities based on PCR amplifications of any gene or DNA fragments must be interpreted with caution. In addition, this study can be applied to the detection of rare transcripts during metatranscriptomic studies or the evaluation of sampling efforts for metagenomics (shotgun sequencing). Improvements in next-generation sequencing technologies together with adequate understanding of biases introduced by the used methodology are the means to achieve realistic estimates of microbial diversity and community structure in environmental systems. Although our current understanding of microbial communities has been greatly enhanced with the introduction of next-generation sequencing technologies, the detection of the complete diversity present in the environment looks still restricted to future developments both on scaling up the processing of higher number of sequences and the limitation of potential biases from the actual community structure.
